# Far upstream element -binding protein 1 (FUBP1) participates in the malignant process and glycolysis of colon cancer cells by combining with c-Myc

**DOI:** 10.1080/21655979.2022.2073115

**Published:** 2022-05-12

**Authors:** Shanwei Wang, Yanli Wang, Sheng Li, Shen Nian, Wenjing Xu, Fenli Liang

**Affiliations:** Department of Pathology, Xi’an Medical College, Xi’an City, Shanxi Province, China

**Keywords:** FUBP1, glycolysis, colon cancer, c-myc

## Abstract

Human distal upstream element (Fuse) binding protein 1 (FUBP1) is a transcriptional regulator of c-Myc and represents an important prognostic marker in many cancers. Therefore, the present study aimed to investigate whether FUBP1 could combine with c-Myc to participate in the progression of colon cancer. Detection of FUBP1 expression was done through reverse transcription-quantitative PCR (RT-qPCR), and the combination of FUBP1 and c-Myc was detected by immunoprecipitation assay. Cell counting kit (CCK)-8, colony formation, transwell and wound healing were applied for assessing the ability of cells to proliferate, migrate, and invade; glycolysis and lactic acid detection kits were used to detect glucose uptake and lactic acid content, while western blotting was adopted to detect the protein expression of glycolysis-related genes. FUBP1 expression was elevated in HCT116 cells relative to other colon cancer cell lines, and silencing FUBP1 could inhibit the ability of HCT116 cells to proliferate, migrate, invade and glycolysis, and enhance its apoptosis. In addition, the results of immunoprecipitation experiments showed that FUBP1 could bind to c-Myc. c-Myc overexpression reversed the inhibitory effects of FUBP1 knockdown on the ability of HCT116 cells to proliferate, migrate, invade and glycolysis. The results indicated that FUBP1 could participate in the deterioration process of colon cancer cells by combining with c-Myc, and it has clinical significance for understanding the key role of FUBP1 in tumor genesis.

## Graphical abstract

**Figure:**
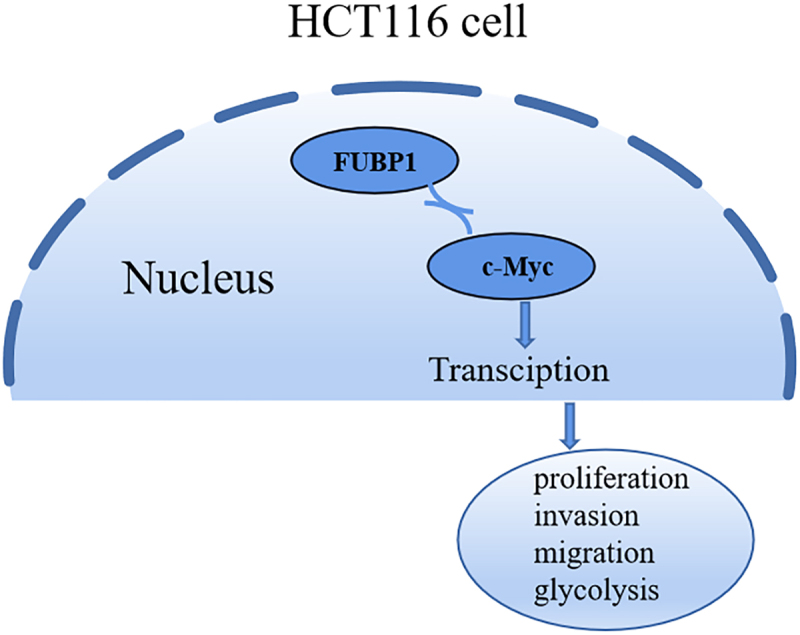

## Highlights

- *Knockdown of FUBP1 can inhibit the invasion, migration, and glycolysis of colon cancer cells.*
- *FUBP1 could bind to c-Myc in colon cancer cells.*
- *Overexpression of c-Myc can promote the proliferation, invasion, migration, and glycolysis of colon cancer cells.*

## Introduction

Colon cancer is recognized as the biggest health killer along with lung cancer, prostate cancer, and breast cancer [1]. Based on cancer statistics published by the American Cancer Society, the incidence of colon cancer in humans is 10.2%, and the mortality rate has reached 9.2%, rising from the fourth to the second [2,3]. In recent years, colon cancer patients are often treated with radical surgical treatment, together with chemoradiotherapy, targeted therapy, gene therapy, and to a large extent improving the survival of patients. However, approximately 50% of colon cancer patients relapse due to drug resistance. About 25% of the patients with colon cancer have liver metastases at the time of initial diagnosis [4,5], and around 50% of the patients experience liver metastases within 3 years after primary surgery [6]. Therefore, studying the pathogenesis of colon cancer is essential to address clinical needs.

With an in-depth understanding of tumor biology and the complexity of tumor metabolism, it has been discovered that metabolic reprogramming is a hallmark of malignant tumors [7,8]. The reason for this is that energy metabolic reprogramming promotes rapid cell growth and proliferation by regulating energy metabolism [9]. Also, the energy metabolism of cancer cells mainly lies in glycolysis, and tumor cells preferentially use glycolysis to produce ATP to achieve aerobic sugar degradation despite sufficient oxygen [10]. Additionally, glucose transport is the first critical step of glycolysis. To meet the demand for energy and intermediates for rapid cell growth, oncogenes often upregulate glucose transporters, especially GLUT1, to increase glucose uptake [11]. Therefore, this reminds us that in some cases, reprogrammed metabolic activities could be used to diagnose, monitor, and treat cancer.

Oncogenes like c-Myc, mTOR, and hypoxia-inducible factor 1α (HIF-1α) are able to increase glycolytic activity to meet anabolic requirements to maintain highly proliferating cancer cells [12–14]. Under normoxic conditions, c-Myc is the ‘master regulator’ of glycolysis and tumor proliferation. c-Myc could transcriptionally up-regulate GLUT1, LDHA, HK2, and PKM2 to promote glycolytic activity, increase glucose uptake and rapid conversion of glucose to lactic acid [15]. Therefore, effective inhibition of c-Myc expression is particularly important to prevent glycolysis and cancer progression.

Human far upstream element (FUSE) binding protein 1 (FUBP1) serves as a major modulator of transcription, translation, and RNA splicing [16]. FUBP1 has been determined to be an effective pro-proliferation and anti-apoptosis factor. For example, FUBP1 can be used as an oncogene to promote the proliferation of oral squamous cell carcinoma and leads to a poor prognosis for patients [17]. It is noted that FUBP1 also can promote the proliferation and deterioration of renal clear cell carcinoma [18]. Additionally, in glioma, FUBP1 effectively facilitates the proliferation of glioma cells, and its mechanism may be related to c-Myc [19]. Jiang et al. reported that FUBP1 could promote the glycolysis of neuroblastoma [20]. It is also found that FUBP1 expression could be increased in colon cancer tissues [21], but there is no study on the specific mechanism.

Therefore, this study hypothesized that FUBP1 was involved in the deterioration processes of colon cancer cells through the combination with c-Myc. The purpose of this work is to analyze FUBP1 expression in colon cancer cells and to explore the impacts of silencing FUBP1 on the ability of colon cancer cells to proliferate, migrate, and invade, as well as apoptosis, glycolysis, and lactic acid production levels, and its internal mechanism.

## Materials and methods

### Cell culture

Human colon cancer cell lines HIEC, LOVO, HCT8, SW620, and HCT116 were provided by the American Type Culture Collection and maintained in RPMI-1640 medium (Thermo Fisher Scientific, Inc.) supplemented with a final concentration of 10% fetal bovine serum and 1% penicillin-streptomycin solution (Beyotime Institute of Biotechnology) in a 5% CO_2_ incubator at 37°C.

### Bioinformatics

The Biogrid DataBase (version 4.4; https://thebiogrid.org/) is a database that can be used to predict the protein-protein link [22].

### Cell transfection

The overexpression of c-Myc (Ov-c-Myc) and the overexpression-negative control (Ov-NC), short hairpin RNA carrying FUBP1 (shRNA-FUBP1, 5’-GCTGCTTATTACGCTCACTAT-3’) and shRNA-Negative Control (shRNA-NC, 5’-TTCTCCGAACGTGTCACGT-3’) were synthesized by Shanghai GeneChem Co., Ltd. HCT116 cells were kept in culture on 12-well plates (3x10^5^ cells/well) in a 5% CO_2_ incubator at 37°C for 24 h. Following incubation, cell transfection was immediately carried out with the aforementioned vectors applying Lipofectamine® 2000 (Invitrogen; Thermo Fisher Scientific) as per the operating procedures of the reagent. The transfection efficiency of genes was evaluated by reverse transcription-quantitative PCR (RT-qPCR) post 48 h [23].

### Cell Counting Kit-8 (CCK-8) assay

Cell inoculation was done in 96-well plates (5x10^3^ cells/well) and continued to incubate for 24, 48, and 72 h at 37°C. Following the incubation, 10 μl of CCK-8 kit was dropped into each well for another 2 h incubation at 37°C. The evaluation of cell viability relied on the absorbance of each well at 450 nm tested with the use of a microplate reader (BioTek, Vermont, USA).

### Wound healing assay

For this experiment, cell inoculation was conducted into 12-well plates (1x10^5^ cells/well). Once HCT116 cells grew to 80% confluence, serum-free RPMI-1640 medium was supplemented into the plates and cultured overnight at 37°C. Subsequently, a single layer of cells was then scratched with a 200-µl pipette tip by a scale. The plates underwent three times of PBS wash and continued to incubate for 24 h at 37°C with 5% CO_2_. Subsequently, the wounds were observed by utilizing a BX51 inverted microscope (Olympus Corporation; magnification, x100). Cell migration was quantified in the following way (0 h scratch width – scratch width following culturing)/0 h scratch width [24].

### Cell invasion assay

A 24-well transwell with 8-μm pore provided by Corning Inc were coated with Matrigel (BD Biosciences) and placed at 37°C for 30 min. 200 μl serum-free medium containing HCT116 cells (5x10^4^ cells/ml) were plated in the upper chamber of transwell and 600 µl RPMI-1640 with 10% FBS was supplemented in the lower chamber for 24-h incubation at 37°C in 5% CO_2_. The cells that failed to cross the membrane were removed by sterile cotton swabs. While the cells that successfully crossed the membrane were fixed in 4% formaldehyde for 15 min. Following staining in 0.1% crystal violet for 30 min at 20°C–25°C, the cells in 5 fields were randomly chosen and observed by an inverted microscope (Olympus Corporation) [25].

### Metabolite measurements

The glucose and lactate concentrations in the medium were determined separately by the Glucose Colorimetric Assay Kit (cat. no. ab136955, Abcam) and Lactate Colorimetric Assay Kit (cat. no. K627-100, Biovision) in light of the manufacturer’s procedures at 48 and 72 h. All metabolite measurements were operated independently no less than three times.

### Extracellular Acidification rate (ECAR) detection

ECAR was measured according to the extracellular flux analyzer (Seahorse Bioscience). Briefly, cells (1.0 × 10^4^ cells/well) were seeded in an XF cell culture plate to achieve 80%–90% confluence. Then, these cells were resuspended in XF assay medium supplemented with 1 mM glutamine (pH = 7.4; Sigma-Aldrich), oligomycin (1 µM; Sigma-Aldrich) and 2-deoxy-D-glucose (2-DG, 100 mM; Sigma-Aldrich). ECAR values were calculated using the program provided by the manufacturer and the data file was exported as a GraphPad Prism file.

### Colony formation assay

HCT116 cells with 5 × 10^2^ cells/well suspended in RPMI-1640 medium were seeded into six-well plates and cultured in a 5% CO_2_ incubator at 37°C for 14 days. Subsequently, these cells were subjected to fixation in 70% ethanol at room temperature for 15 min and staining in 0.05% crystal violet for 20 min at 37°C. The number of colonies formed (>50 cells/colony) was calculated by counting with an Olympus BX40 light microscope (Olympus Corporation) [26].

### Co-immunoprecipitation assay

About 5 × 10^7^ cells were lysed with Triton X-100 lysis buffer (40 mM Tris, 1% Triton X-100, 1 mM NaF, 1 mM Na_3_VO_4_) supplemented with protease inhibitor cocktail to obtain cell lysates. Following determination of total protein concentrations by BCA assay, rabbit anti-FUBP1 polyclonal antibody was used to incubate with protein A beads at 4°C for 1 h, and then these beads were added with 450 μM DSS solution following the manufacturer’s protocol. Next, FUBP1 and its interacting proteins were purified with the antibody conjugated beads, followed by mass spectrometric analysis or western blotting [27].

### RT-qPCR analysis

Whole RNA extraction was undertaken from HCT116 cells by means of a TRIzol® reagent (Thermo Fisher Scientific), followed by a reverse transcription to cDNA with the employment of FastQuant RT kit (cat. no. KR106; Tiangen Biotech Co., Ltd.) in strict accordance with the maker’s protocols. qPCR reactions were conducted adopting the PowerUp™ SYBR™ Green Master Mix (cat. no. A25779; Applied Biosystems) on the ABI 7500 PCR system (Applied Biosystems). The thermocycling conditions were described below: initial denaturation at 94°C for 30 sec, followed by 16 cycles at 55°C for 30 sec and 72°C for 30 sec. The relative expressions of genes were normalized to those of the housekeeping gene GAPDH and were calculated utilizing the 2^−ΔΔCq^ method [28]. The sequences of all genes were listed below: FUBP1 forward, 5’-CAACCAGATGCTAAGAAAGTTGC-3’ and reverse, 5’-CCTCCTCTGCCAATTATGAATCC-3’; GLUT-1 forward, 5’-TAGTACTGGGTGGCAGA-3’ and reverse, 5’-CGGCACAAGAATGGATGAAA-3’; PKM2 forward, 5’-AAGGGTGTGAACCTTCCTGG-3’ and reverse, 5’-GCTCGACCCCAAACTTCAGA-3’; LDAGCAGHA forward, 5’-TGATGGATCTCCAACAGCAGATGG-3’ and reverse, 5’-CAGCTTGGAAGCAGGTTTGCAGTTAC-3’; c-Myc forward, 5’-GGCTCCTGGCAAAAGGTCA-3’ and reverse, 5’-CTGCGTAGTTGTGCTGATGT-3’; GAPDH forward, 5’-GATGATGTTGAACTCGTCGC-3’ and reverse, 5’-CTCTTCTGGGTTTCTCACACC-3’.

### Western blotting

HCT116 cells treated in each group were collected and added with RIPA buffer (Beyotime Biotechnology Institute) to extract total proteins, which were then detected by applying BCA protein Detection Kit (Beyotime Biotechnology Institute) for concentration determination. The protein samples was transferred to PVDF membranes soaked in methanol and sealed with 5% bovine serum albumin at room temperature for 30 min. The membranes were mixed with primary antibodies against FUBP1 (cat. no. ab189914; 1:2000 dilution; Abcam), GLUT-1 (cat. no. ab92742; 1:1000 dilution; Abcam), PKM2 (cat. no. ab85555; 1:1000 dilution; Abcam), LDHA (cat. no. ab134187; 1:2000 dilution; Abcam), c-Myc (cat. no. ab32072; 1:1000 dilution; Abcam); matrix metallopeptidase (MMP)2 (cat. no. ab215986; 1:1000 dilution; Abcam); MMP12 (cat. no. ab52897; 1:1000 dilution; Abcam) and GAPDH (cat. no. ab181602; 1:10,000 dilution; Abcam) at 4°C overnight. The next day, the secondary antibodies conjugated to horseradish peroxidase (1:5000; Santa Cruz Biotechnology, Inc.) were added into the membranes for another 2 h incubation at room temperature. After adding ECL luminescent solution, the protein bands were obtained by the gel imager (C150, Azure Biosystems, USA). The gray value of the protein bands was subjected to analysis employing ImageJ (v1.51) software and the relative expression of the protein was calculated [29].

### Statistical analysis

The results of all the experiments were used for data analysis with GraphPad Prism 7 (GraphPad Software, Inc.). These figures from three independent experiments were presented here in the form of mean ± standard deviation. Comparisons between two groups were made utilizing an unpaired Student’s t-test, while comparisons of those among multiple groups were done by means of one-way ANOVA followed by Tukey’s post hoc test. A P value <0.05 for each group was determined to be statistically significant.

### Results

This study investigated whether FUBP1 is involved in the deterioration processes of colon cancer cells through the combination with c-Myc. First, we evaluated the effects of FUBP1 on proliferation, invasion, migration, and glycolysis of colon cancer cells. We found that knockdown of FUBP1 abviously inhibited proliferation, invasion, migration, and glycolysis of HCT116 cells. Subsequently, we further evaluated whether c-Myc was involved in the mechanism of FUBP1 in colon cancer. The results showed that FUBP1 could bind to c-Myc and overexpression of c-Myc blocked the interference of FUBP1 and promoted the proliferation, invasion, migration, and glycolysis of colon cancer cells.

**Figure 1.**
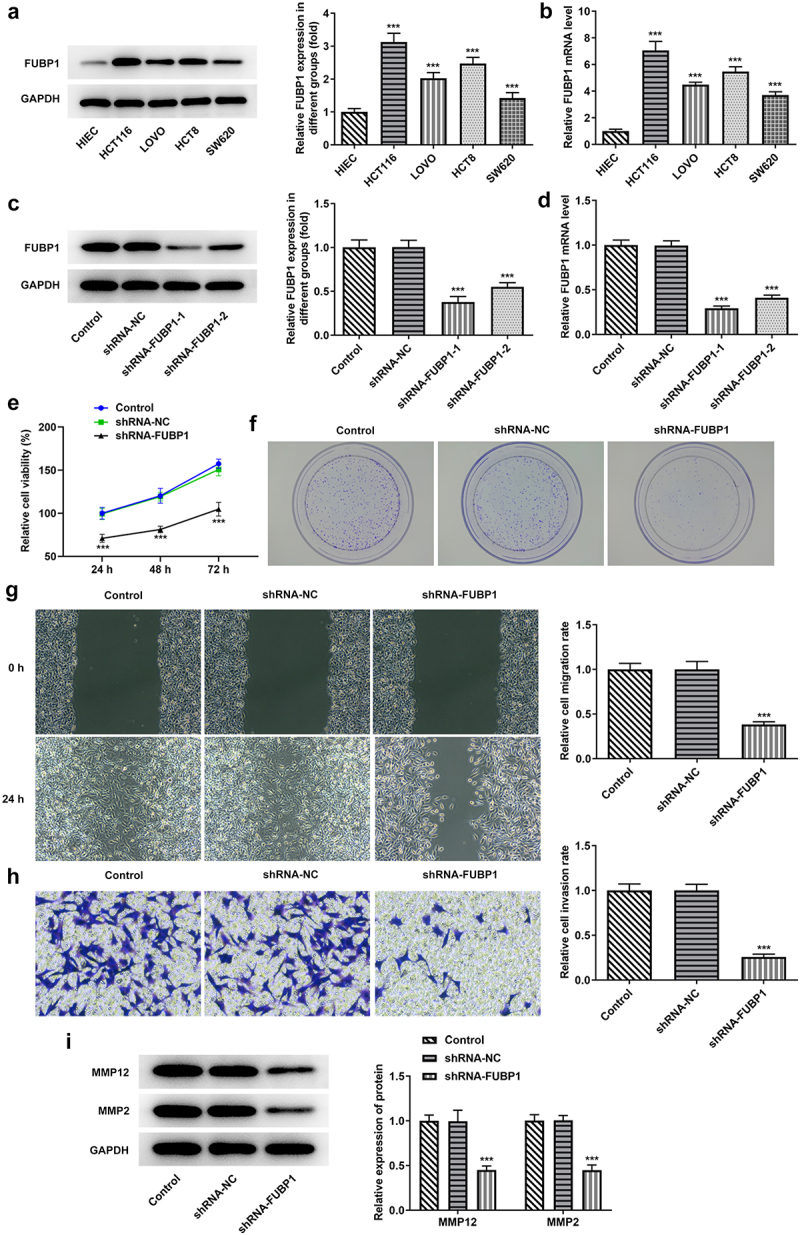
**FUBP1 expression level is increased in colon cancer cell lines, and knockdown FUBP1 inhibits colon cancer cell proliferation, invasion and migration**. FUBP1 protein (a) and mRNA (b) expressions in colon cancer cells. FUBP1 protein (c) and (d) mRNA expressions following FUBP1 knockdown in HCT116 cells. ***p < 0.001 vs. HIEC or control; (e) Cell proliferation was assessed by performing a Cell Counting Kit-8 assay. (f) Representative images of colony formation assay. (g) Representative images of wound healing assay (magnification, x100). (h) Representative images of Transwell assay (magnification, x100). (i) MMP2 and MMP12 protein expressions were assessed via western blotting. ***P < 0.001 vs. shRNA-NC. FUBP1, Human far upstream element (FUSE) binding protein 1; MMP, matrix metallopeptidase; shRNA, short hairpin RNA; NC, negative control.

*FUBP1 is upregulated in colon cancer cell lines and knockdown of FUBP1 inhibits HCT116 cell invasion and migration*. First, the expression of FUBP1 was analyzed by western blotting and RT-qPCR in colon cancer cell lines. Compared with other colon cancer cell lines (HIEC, LOVO, HCT8, SW620), HCT116 cell line exhibited the highest FUBP1 expression (Figures 1a-b). Thus, this cell line was utilized for later experiments. Additionally, HCT116 cells with shRNA-FUBP1-1 showed lower expression than those of shRNA-FUBP1-2, therefore, shRNA-FUBP1-1 was chosen for following biological function experiments (Figures 1c-d). After that, cell viability in HCT116 cells was estimated by CCK-8 and colony formation assays. It was easily seen that FUBP1 knockdown repressed HCT116 cell proliferation (Figures 1e-f). Subsequently, wound healing and transwell experiments were used to evaluate the ability of invasion and migration in HCT116 cells after shRNA-FUBP1-1 transfection. In contrast to the NC groups, the cell invasion and migration ability was found to be weakened in the shRNA-FUBP1-1 group (Figures 1g-h). Furthermore, the levels of invasion and migration-related proteins (MMP2 and MMP12) were also detected. The results indicated that MMP2 and MMP12 levels were significantly decreased in the shRNA-FUBP1 group compared with the shRNA-NC group (Figure 1i). These results indicated that FUBP1 was expressed at a high level in HCT116 cell, and its knockdown could suppress the ability of HCT116 cells to proliferate, migrate, and invade.

**Figure 2.**
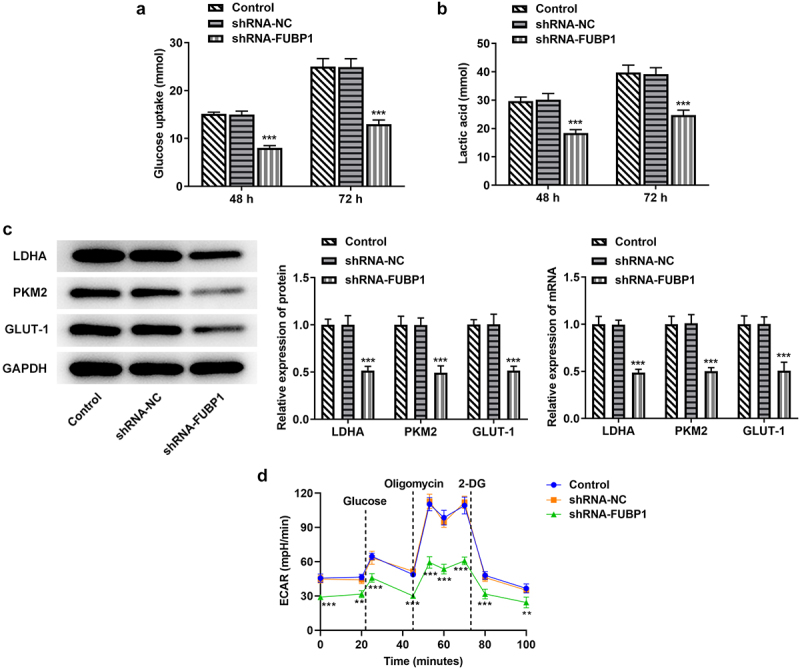
**FUBP1 knockdown alleviates glycolysis in colon cancer cells**. (a) The glucose content in HCT116 cells was detected by glucose detection kit. (b) The lactic acid kit was utilized to measure the level of lactic acid in the HCT116 cells. (c) The expressions of glycolytic associated proteins (GLUT-1, PKM2 and LDHA) were detected using RT-qPCR and western blotting. (d) The ECAR was detected as an indicator for deduced glycolysis flux and glycolytic capacity. *P < 0.05, **P < 0.01 and ***P < 0.001 vs shRNA-NC. FUBP1, Human far upstream element (FUSE) binding protein 1; shRNA, short hairpin RNA; NC, negative control; ECAR, extracellular acidification rate.

*Knockdown of FUBP1 represses glycolysis in HCT116 cells*. The glucose uptake detection kit was used to evaluate the glycolytic ability of HCT116 cells after FUBP1 inhibition. The results showed that glucose intake was declined in the shRNA-FUBP1 group at 48 h or 72 h (vs sh-NC; Figure 2a). Lactic acid detection kit was used to detect the production of lactic acid in HCT116 cells, it was found that the content of lactic acid in HCT116 cells was also decreased after FUBP1 was inhibited (Figure 2b). Besides, the expression of glucose degradation-related genes (LDHA, PKM2, and GlUT-1) were also tested. RT-PCR and western blotting results showed that the expressions of these genes was declined. Therefore, the above results all indicate that the glycolysis ability of HCT116 cells was significantly reduced after transfection with shRNA-FUBP1-1 (Figure 2c). Additionally, we analyzed extracellular acidification rate (ECAR), the steady state glycolysis flux, and glycolytic capacity were attenuated in shRNA-FUBP1 group, indicating that FUBP1 can promote glycolysis flux (Figure 2d).

**Figure 3.**
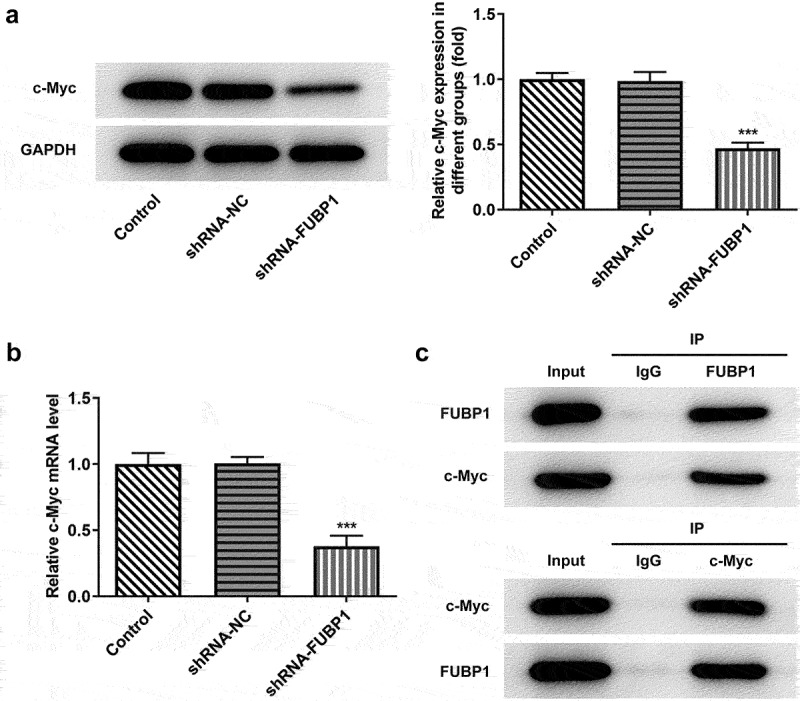
**FUBP1 binds to c-Myc in colon cancer cells**. c-Myc protein (a) and (b) mRNA expressions following FUBP1 knockdown in HCT116 cells. (c) Co-IP assay was applied to detect the combination of FUBP1 and c-Myc. ***P < 0.001 vs. shRNA-NC. FUBP1, Human far upstream element (FUSE) binding protein 1; shRNA, short hairpin RNA; NC, negative control.

*FUBP1 could bind to c-Myc in colon cancer cells*. Subsequently, the present study further investigated the mechanism underlying the effects of FUBP1 on HCT116 cells. Bioinformatics analysis based on the Biogrid database (https://thebiogrid.org/) suggested that FUBP1 could bind to c-Myc. c-Myc, as a tumor-promoting gene, is involved in the progression of a variety of tumors. Therefore, there might be some potential mechanism between FUBP1 and c-Myc in the pathogenesis of colon cancer. Interestingly, it was found that c-Myc expression was significantly downregulated in HCT116 cells transfected with shRNA-FUBP1 (Figures 3a-b), indicating that FUBP1 was linked to c-Myc expression. To further determinate the association, we adopted COIP experiment to detect the binding between FUBP1 and c-Myc. As seen in Figure 3c, the precipitation experiment with IgG showed that FUBP1 protein and c-Myc protein were not precipitated, which indicated that FUBP1 protein and c-Myc protein could not bind to IgG. Notably, we found that both protein c-Myc and protein FUBP1 could be precipitated either by using protein FUBP1 to precipitate protein c-Myc or by using protein c-Myc to precipitate FUBP1. These results indicated that there was an exact interaction between FUBP1 and c-Myc.

**Figure 4.**
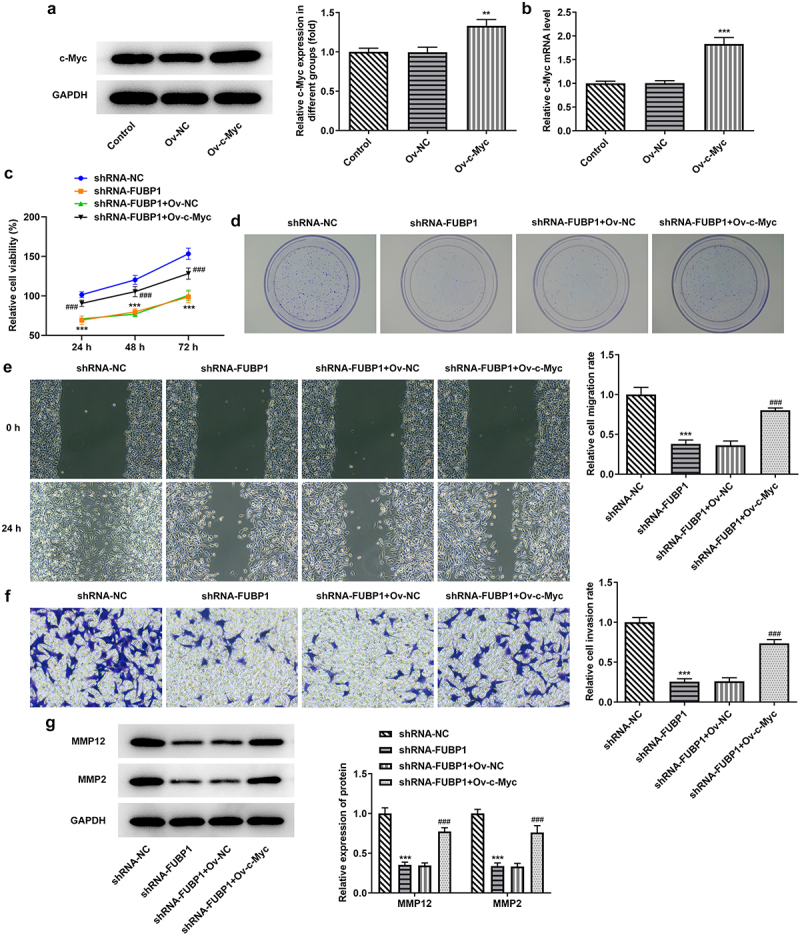
**Overexpression of c-Myc could promote the proliferation, invasion and migration of colon cancer cells transfected with shRNA-FUBP1**. c-Myc protein (a) and mRNA (b) expressions following c-Myc overexpression in HCT116 cells. (c) Cell proliferation was assessed by performing Cell Counting Kit-8 assays. (d) Representative images of colony formation assay. (e) Representative images of wound healing assay (magnification, x100). (f) Representative images of Transwell assay (magnification, x100). MMP2 and MMP12 protein expressions were measured via western blotting. *P < 0.05, **P < 0.01 and ***P < 0.001 vs Ov-NC or shRNA-NC; ^##^P < 0.01 and ^###^P < 0.001 vs. shRNA-FUBP1 + Ov-NC; FUBP1, Human far upstream element (FUSE) binding protein 1; shRNA, short hairpin RNA; Ov, overexpression; NC, negative control.

*Overexpression of c-Myc blocks the interference of FUBP1 and promotes the proliferation, invasion, and migration of colon cancer cells*. To elucidate whether c-Myc could bind with FUBP1 to participate in the malignant process of colon cancer cells, a plasmid overexpressing c-Myc (Ov-c-Myc) was constructed and transfected into HCT116 cells. The expression of c-Myc in the Ov-c-Myc group was successfully increased compared with that in the Ov-NC group (Figures 4a-b). Additionally, the overexpression of c-Myc facilitated cell viability and colony formation in HCT116 cells transfected with shRNA-FUBP1+ Ov-c-Myc (Figures 4c-d). Results from wound healing and transwell assay revealed that c-Myc overexpression also promoted cell invasion and migration ability of HCT116 cells following the FUBP1 knockdown (Figures 4e-f). Moreover, the expressions of MMP2 and MMP12 were discovered to be elevated in shRNA-FUBP1+ Ov-c-Myc group (Figure 4g).

**Figure 5.**
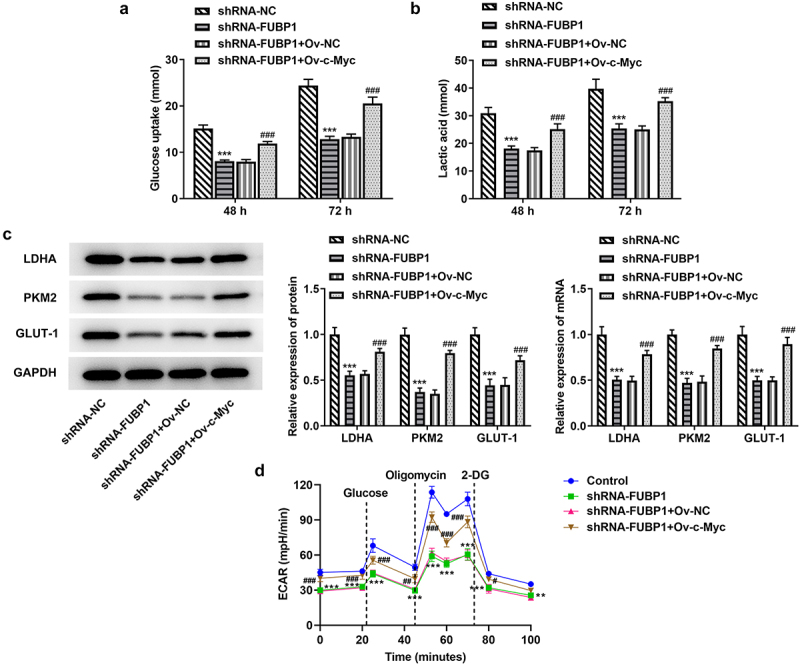
**Overexpression of c-Myc could promote the glycolysis of colon cancer cells**. (a) Glucose content was detected by glucose detection kit. (b) Lactic acid level was measured by lactic acid kit. (c) The expressions of glycolytic associated proteins (GLUT-1, PKM2 and LDHA) were detected using RT-qPCR and western blotting. *P < 0.05, **P < 0.01 and ***P < 0.001 vs shRNA-FUBP1; (d) The ECAR was detected as an indicator for deduced glycolysis flux and glycolytic capacity. ^##^P < 0.01 and ^###^P < 0.001 vs. shRNA-FUBP1 + Ov-NC, Human far upstream element (FUSE) binding protein 1; shRNA, short hairpin RNA; Ov, overexpression; NC, negative control; ECAR, extracellular acidification rate.

*Overexpression of c-Myc could block the interference of FUBP1 and promote the Glycolysis of colon cancer cells*. A glucose uptake kit was used to detect the glucose uptake capacity of HCT116 cells co-transfected with shRNA-FUBP1 and Ov-c-Myc. Overexpression of c-Myc increased the glucose uptake ability of HCT116 cells co-transfected with shRNA-FUBP1 and Ov-c-Myc at 48 h and 72 h (Figure 5a). Furthermore, the level of lactic acid was remarkably elevated at 48 h and 72 h in HCT116 cells co-transfected with shRNA-FUBP1 and Ov-c-Myc (Figure 5b). Meanwhile, the expressions of LDHA, PKM2, and GlUT1 related to glycolysis were also upregulated following HCT116 cells were transfected with shRNA-FUBP1+ Ov-c-Myc at 72 h (Figure 5c), furthermore, ECAR detection showed that c-Myc overexpression reversed the inhibition of FUBP1 interference on glycolysis (Figure 5d). These results collectively suggested that c-Myc could bind to FUBP1, and ultimately promote the malignant progression and glycolysis level of colon cancer cells.

## Discussion

The function of FUBP1 in various cancers raises growing interest. FUBP1 is involved in the regulation of many cellular processes, including gene expression and cell differentiation [30–32]. The existing body of research suggest that FUBP1 protein level is increased in human osteosarcoma cell line [33]. Engidwork et al. [34] found similarly increased level of FUBP1 in human medulloblastoma cell lines. In addition, a study on digestive tract tumors showed that FUBP1 directly or indirectly induced tumor cell proliferation in the liver cancer cell cycle [35]. As mentioned, FUBP1 is a potential cancer-promoting regulator in a variety of tumors. In this study, we observed that there was an elevated expression of FUBP1 in colon cancer cells. Not only that, in vitro cell experiments showed that shRNA-FUBP1 could effectively inhibit the proliferation and migration of HCT116 cells. And declined glycolysis level implied that FUBP1 knockdown also inhibited tumorigenesis and development of colon cancer cells.

Notably, the relevance of FUBP1 and c-Myc expression in several cancers suggests that FUBP1 may play a role in the carcinogenesis process by depending on the c-Myc pathway. For instance, loss of FUBP1 function leads to decreased c-Myc expression and cell proliferation [36]. Weber et al. [37] also noted that FUBP1 protein is associated with c-Myc expression in clear cell renal carcinoma, and results in poor prognosis. Moreover, Ding et al. [19] found that c-Myc was expressed in more than 75% of gliomas, and its high expression was also related to the low survival rate of human gliomas. Jin et al. showed that c-Myc knockout could inhibit the occurrence and metastasis of triple negative breast cancer by reducing the expression of Hsa-mir-4723-5p [38]. Treating c-Myc-dependent tumors could be achieved by affecting the expression of FUBP1 [16]. Therefore, this study aimed at identifying the interactions and molecular mechanisms between FUBP1 and c-Myc in colon cancer cells. First, the results of Biogrid database and COIP experiment showed that FUBP1 could target with c-Myc. After silencing FUBP1 and overexpressing c-Myc, the malignant biological behaviors and glycolysis was observed in colon cancer cells. These findings indicated that FUBP1 exerted an essential role in colon cancer. Previous findings suggest that FUBP1 may play a role in the carcinogenesis process by relying on the c-Myc pathway [39]. Compared with the results of Jin et al. [38]. This study further identified the important role of FUBP1 binding to c-Myc in colon cancer progression.

In this study, we looked at the impact of FUBP1 binding to c-Myc on colon cancer cells. Further studies are needed to explore other mechanisms of FUBP1 in colon cancer cells. Furthermore, only in vitro experiments were done in the present study, and the in vivo experiments have not been further confirmed. Therefore, the effects and mechanisms of FUBP1 on colon cancer should be studied in animal models in the future.

## Conclusion

To sum up, this study was the first to suggest that the inhibition of malignant biological behavior and the promotion of glycolysis by FUBP1 in colon cancer cells was related to c-Myc, which would provide a reference for determining the potential mechanism of FUBP1 in the development of colon cancer.

## Data Availability

The datasets used and/or analyzed during the current study are available from the corresponding author on reasonable request.
